# Epidemiological and clinical characteristics of the largest COVID-19 outbreak along the China-Myanmar border in Ruili City, Yunnan Province, China

**DOI:** 10.3389/fpubh.2022.962214

**Published:** 2022-08-23

**Authors:** Xiangyu Yan, Linhui Hao, Zekun Wang, Xuechun Wang, Xiangyu Zhang, Tao Li, Zhongwei Jia, Litao Chang, Bo Zhang, Tiejun Shui

**Affiliations:** ^1^School of Public Health, Peking University, Beijing, China; ^2^Yunnan Center for Disease Control and Prevention, Kunming, China; ^3^Chinese Center for Disease Control and Prevention, Beijing, China; ^4^Center for Intelligent Public Health, Institute for Artificial Intelligence, Peking University, Beijing, China; ^5^Center for Drug Abuse Control and Prevention, National Institute of Health Data Science, Peking University, Beijing, China; ^6^Peking University Clinical Research Institute, Beijing, China

**Keywords:** China-Myanmar border, clinical characteristics, COVID-19 vaccination, outbreak, SARS-CoV-2 Delta VOC, transmission

## Abstract

**Background:**

Imported COVID-19 patients posed great challenges to border areas' COVID-19 control. However, research was scarce to reveal epidemiological characteristics of COVID-19 in border areas. This study aimed to explore the detailed transmission chains, and reveal epidemiological and clinical characteristics of the largest COVID-19 outbreak caused by Delta variant of concern (VOC) occurred in the China-Myanmar border area.

**Methods:**

During the outbreak from July to September, 2021 in Ruili City, Yunnan Province, China, epidemiological investigation data and clinical-related data pertaining to confirmed COVID-19 patients were collected. Patients' contact history data and viral gene sequencing were used for inference of transmission chains. Sociodemographic and epidemiological characteristics, cycle threshold (Ct) value, and antibodies level were compared between patients who were vaccinated against COVID-19 or not.

**Results:**

A total of 117 COVID-19 patients were confirmed during the outbreak, among which 86 (73.5%) were breakthrough infections. These patients evenly split between Chinese and Myanmar people (50.4% vs. 49.6%). Most of these patients were mild (45.3%) or moderate (48.7%) infections with no death reported. Multi-source of infection led to 16 transmission chains with a maximum of 45 patients in one chain. Patients vaccinated against COVID-19 before infection had relatively higher antibodies (IgM and IgG) levels and more rapid response to infection than non-vaccinated patients (*p* < 0.05).

**Conclusion:**

Land border areas have greater risks of imported COVID-19 and more complicated epidemics. It should be cautious in formulating entry and exit requirements for border areas. The immune effect of COVID-19 vaccines and related mechanism should be further explored.

## Introduction

The ongoing coronavirus disease 2019 (COVID-19) epidemic has led to a high burden in public health and people's daily lives. After the highly infectious SARS-CoV-2 Delta variant strain (B.1.617.2) was first found in India, it spread rapidly around the world until now and was listed as one of the two currently circulating variants of concern (VOCs) by World Health Organization (WHO) (1). Although the Omicron VOC is now the dominant strain in many countries, a recent study has indicated that the Omicron has not eliminated Delta VOC and the Delta might have the risk to re-emerge (2). Considering the relatively higher infectivity and transmissibility of the Delta VOC (3), it still deserves enough attention.

Since the first local Chinese COVID-19 patient infected with SARS-CoV-2 Delta VOC was detected in Guangzhou, China on 21 May 2021, Delta VOC has spread to more than 50 cities around China and led to eleven outbreaks, all of which were caused by imported COVID-19 patients (4). The pressure of COVID-19 prevention and control brought by imported cases in border areas was even greater. China has 14 land neighbors, and the China-Myanmar border in the southwest is a typical representative of land borders. The border area is near the Kokang region in northern Shan State of Myanmar that are often at civil war, which results in poor accessibility to basic health services (5). In addition, represented by Jiegao Border Trade Zone, the commodity trade between China and Myanmar is prosperous in this area, which is also China's main jade raw material distribution center and a popular tourist destination. Because of the above geographical and cultural characteristics, the China-Myanmar border area in China is a traditional area with high risk of imported infectious diseases, such as malaria (6), which also faces the a huge pressure caused by imported COVID-19 cases and the risk of the epidemic spreading across the country during the COVID-19 pandemic. However, few studies have revealed the detailed epidemiological characteristics of COVID-19 in border areas.

On 4 July, 2021, three confirmed COVID-19 patients infected with Delta VOC was reported by the government of Ruili City, Yunnan Province, since then, the largest COVID-19 outbreak caused by Delta VOC in China's border areas occurred, which was named as the “July 4” COVID-19 outbreak. In this study, we aimed to explore the detailed transmission chains, and reveal epidemiological and clinical characteristics of this outbreak occurred in the China-Myanmar border areas.

## Methods

### Study design and epidemiological survey

**Figure 1:**
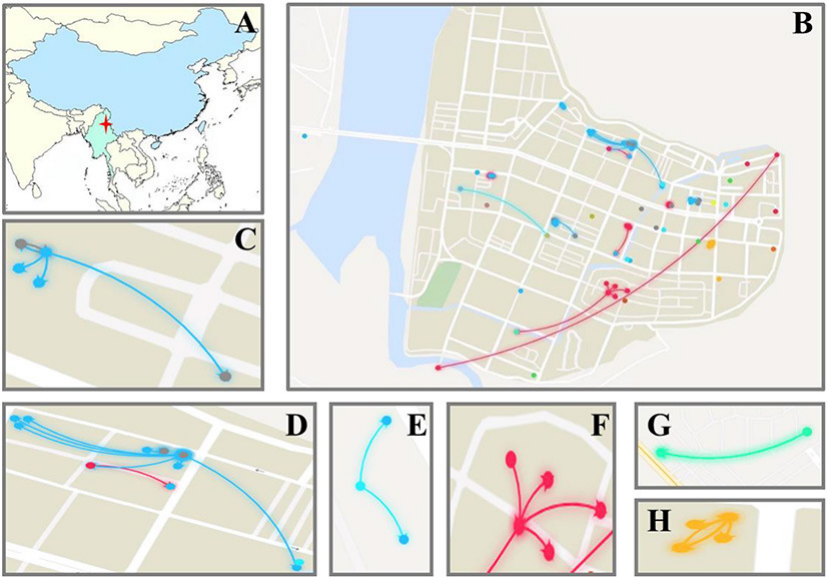
Geographical distribution of confirmed patients and transmission of the virus. **(A)**: The location of Ruili City. **(B–H)**: Geographical distribution of confirmed cases and transmission chains; the lines mean the virus transmission caused by close contacts; different colors of lines means different transmission chains.

All confirmed patients infected with SARS-CoV-2 Delta VOC during the “July 4” outbreak from July to September, 2021 in Ruili City, Yunnan Province, China, which is an important land border port city near the China-Myanmar border (Figure 1A), were included in this study. According to the National Health Commission's guidelines (7), the diagnosis of COVID-19 patients were confirmed by the local Center for Disease Control and Prevention (CDC), and were classified into mild, moderate, severe, and critical according to severity. Detailed epidemiological investigation and clinical data collection were conducted to confirmed patients and their close contacts by local CDC and the Yunnan Provincial CDC.

Based on the epidemiological investigation, these patients' sociodemographic information, address where the patients might be infected (residential and/or workplace address), travel track, contact history, and other activities information were collected. These patients' close contacts were found based on epidemiological investigation data and travel history big data. The detailed epidemiological investigation was also conducted to the close contacts. Using the unique identifier making up of ID number and name, their COVID-19 vaccination records were extracted from the vaccination database by the local CDC's staff.

The ethical approval was provided by the Mang City Center for Disease Control and Prevention, Yunnan, China, and all participants gave oral informed consent because of their level of education and the urgency of emerging infectious disease's control.

### Laboratory test

Regular nasopharyngeal swabs of confirmed COVID-19 patients were collected for SARS-CoV-2 nucleic acid testing using Reverse Transcriptase-Polymerase Chain Reaction (RT-PCR) targeting of ORF1ab and N genes. The Novel Coronavirus 2019 Nucleic Acid Test Kit (manufactured by Bojie Medical Technology, Shanghai Municipality and Daan Gene Company, Guangzhou City, China). A cycle threshold (Ct) value of below 40 was considered as a positive result. Regular serum samples were collected from confirmed COVID-19 patients at least once a week for antibodies IgM and IgG testing. Anti-SARS-CoV-2 Rapid Test Kit (manufactured by Antubio Diagnostics Company, Zhengzhou, China) was used, and a value of over 1 S/CO was considered as positive result. The biological samples of these patients were also sent to the China CDC to obtain virus genotyping and inference of transmission chains. The information of transmission chains used in this study was obtained from China CDC's reports combining with the contacts history among patients. The complete genomic sequences were acquired from swab samples. Compared with the reference genome sequence of Wuhan (GenBank No.NC_045512), genome sequences from Ruili's patients existed 35–45 unique nucleotide mutation sites, which belonged to Delta variant strain (B.1.617.2). Based on the phylogenetic tree analysis, the genome sequences of Ruili's patients could be assigned to different lineages, and patients in the same lineages could be considered as the same transmission chains. The methods of genomic sequences analysis were described in previous studies in detail (8–10).

### Outbreak control measures

In order to effectively control the outbreak, the following measures were implemented. First, patients were immediately transported to a designated hospital for treatment and quarantine, and their close contacts received medical observation at designated hotels or home for 14 days. Second, epidemiological investigations were conducted to confirmed COVID-19 patients and their close contacts to know details about the history of travel, work, contacts, and activities; and regular nucleic acid testing and serum SARS-CoV-2 antibody testing were provided to them. COVID-19 patients with two consecutive negative nucleic acid test results and without COVID-19-related symptoms, signs and imaging abnormalities could be discharged. For their close contacts, the medical observation could also be lifted for those who had no typical symptoms or positive nucleic acid test results during the 14 days. Third, regular SARS-CoV-2 nucleic acid testing for high-risk populations was conducted, and regular disinfection of public places, medical quarantine sites, and border areas were strengthened. Fourth, the administration of entry and exit in border areas was also strengthened, and all people entering China needed to be retested for SARS-CoV-2 nucleic acid regularly. Because of these proactive and positive measures, COVID-19 did not spread widely and the “July 4” outbreak was declared over on 15 September 2021.

### Statistical analysis

The confirmed COVID-19 patients were divided into two groups according to whether they were vaccinated against COVID-19. Sociodemographic and epidemiological characteristics, Ct value, and antibodies level were described and compared between the two groups. Student's *t* test or Mann-Whitney *U* test was used to compare continuous variables when applicable, respectively. Categorical variables were compared by chi-square test or Fisher's exact test when data was limited. A p value of 0.05 or less was regarded as significant difference for the two-sided test. Statistical analyses were done with SPSS version 21.0 (IBM Corp).

## Results

**Figure 2:**
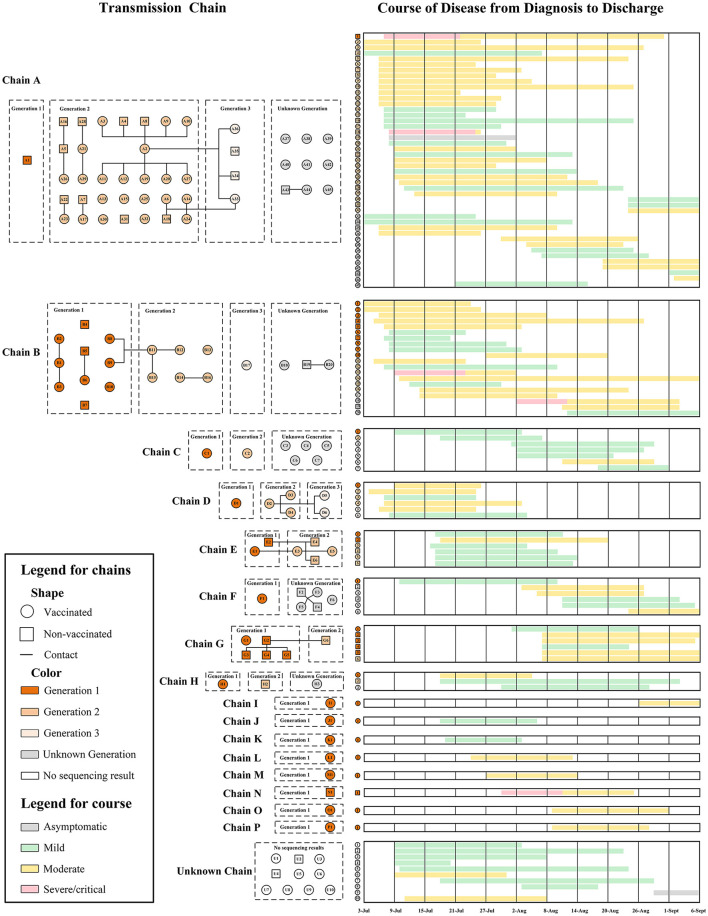
Transmission chains and confirmed patients' course of disease during the outbreak.

After the first three cases (index cases, including two Chinses men and one Myanmar man) were reported, two waves of cases were observed in the outbreak, of which the largest wave was in early July, and the other was in early August. After that, cases were sporadic until the outbreak ended in September (Supplementary Figure 1). A total of 117 COVID-19 patients were confirmed in the “July 4” outbreak with an average age of 34.01 ± 16.18 years, among which 86 (73.5%) were breakthrough infections with a median of 65 days (IQR: 34–159 days) from the date of the last dose of vaccine to the first positive nucleic acid test result. Most of these patients (65.0%) were 18–45 years and were evenly split between male (51.3%) and female (48.7%). About a half of them (49.6%) came from Myanmar, and 58.1% of them reported that they had clear contact history with other COVID-19 patients. Most of these patients were mild (45.3%) or moderate (48.7%) infections with no death in this outbreak (Table 1, Figure 2).

**Table 1:** Characteristics of the 117 confirmed COVID-19 patients during the outbreak.

**Characteristics**	**Total** **(*N* = 117)** ***N* (%)**	**Vaccinated** **(*N* = 86)** ***N* (%)**	**Non-vaccinated** **(*N* = 31)** ***N* (%)**	***P* value**
Age (years)			
Mean ± SD	34.01 ± 16.18	36.37 ± 13.42	27.45 ± 21.03	0.033
<18	12 (10.3)	0 (0.0)	12 (38.7)	<0.001
≥ 18 to <45	76 (65.0)	64 (74.4)	12 (38.7)	
≥ 45 to <65	24 (20.5)	19 (22.1)	5 (16.1)	
≥65	5 (4.3)	3 (3.5)	2 (6.5)	
Sex				0.834
Male	60 (51.3)	45 (52.3)	15 (48.4)	
Female	57 (48.7)	41 (47.7)	16 (51.6)	
Nationality				0.067
China	59 (50.4)	39 (45.3)	20 (64.5)	
Myanmar	58 (49.6)	47 (54.7)	11 (35.5)	
Contact history with COVID-19 patients				0.091
Yes	68 (58.1)	46 (53.5)	22 (71.0)	
No	49 (41.9)	40 (46.5)	9 (29.0)	
Case classification				0.261
Asymptomatic	2 (1.7)	2 (2.3)	0 (0.0)	
Mild	53 (45.3)	38 (44.2)	15 (48.4)	
Moderate	57 (48.7)	44 (51.2)	13 (41.9)	
Severe/critical	5 (4.3)	2 (2.3)	3 (9.7)	
Ct value on admission [median (IQR)]				
ORF1a/b gene	19.71 (16.34–25.69)	19.71 (16.25–26.20)	19.71 (16.42–24.33)	0.973
N gene	18.14 (14.66–25.08)	18.09 (14.99–25.64)	18.59 (13.45–22.16)	0.626
Antibodies test on admission				
IgM (S/CO)			
median (IQR)	0.07 (0.03–0.16)	0.09 (0.05–0.23)	0.02 (0.00–0.05)	<0.001
Positive	8 (6.8)	8 (9.3)	0 (0.0)	0.108
Negative	109 (93.2)	78 (90.7)	31 (100.0)	
IgG (S/CO)		
median (IQR)	1.19 (0.05–3.58)	2.12 (0.76–4.31)	0.01 (0.00–0.05)	<0.001
Positive	61 (52.1)	61 (70.9)	0 (0.0)	<0.001
Negative	56 (47.9)	25 (29.1)	31 (100.0)	

Based on gene sequence inference, 16 transmission chains were identified, of which the largest (Chain A) included 45 patients and the virus transmitted for three generations. There were also eight transmission chains (Chain I–P) that had only one patient (Figure 2). Nine of the 16 transmission chains included Myanmar patients, and the index cases (Generation 1 cases) in five of these transmission chains were confirmed to come from Myanmar. Majority of the transmission chains were in Jiegao Community, which was the largest land port and trade zone between China and Myanmar (Figure 1B). The address where the patients might be infected and the geographic connections of patients in different transmission chains were shown in Figures 1B–H. Some clustered transmissions in adjacent buildings or stories were observed in this outbreak (Figures 1E–H). Meanwhile, because of the transportation and the need of goods delivery, virus could also spread over longer distances across blocks (the red lines in Figure 1B, and the blue lines in Figures 1B–D).

Patients who had been vaccinated against COVID-19 were older than non-vaccinated patients (p <0.05), and 38.7% of the non-vaccinated patients were children under 18 years, while no children infections were found among vaccinated patients (*p* < 0.05). On admission, the IgM and IgG values of vaccinated patients were higher than that of non-vaccinated patients (*p* < 0.05), 70.9% of vaccinated patients had positive IgG test results while no patient's IgG test result was positive among non-vaccinated patients (*p* < 0.05) (Table 1). During the five-week course of disease after diagnosis, the antibodies values of vaccinated patients were still higher than non-vaccinated (*p* < 0.05). In addition, the antibodies rose more quickly among vaccinated patients than non-vaccinated, which showed a sharper increase in the second week of both IgM and IgG among vaccinated patients, while the sharpest increase was observed in the third week of non-vaccinated patients (Table 2).

**Table 2:** Antibody values in the course of disease after diagnosis during the outbreak.

**Antibody**	**Week**	**Total (*N* = 117)** **median (IQR)**	**Vaccinated (*N* = 86)** **median (IQR)**	**Non-vaccinated (*N* = 31)** **median (IQR)**	***P* value**
IgM (S/CO)				
	Week 1	0.20 (0.05–0.56)	0.33 (0.11–0.74)	0.03 (0.02–0.07)	<0.001
	Week 2	1.78 (0.86–6.75)	2.89 (1.30–10.01)	0.40 (0.18–1.13)	<0.001
	Week 3	1.80 (0.93–6.90)	3.38 (1.05–9.32)	1.17 (0.37–2.16)	0.001
	Week 4	1.92 (0.71–5.98)	2.72 (0.83–6.82)	0.77 (0.21–2.47)	0.004
	Week 5	1.94 (0.63–4.52)	2.18 (1.35–5.40)	0.80 (0.31–2.38)	0.017
IgG (S/CO)				
	Week 1	2.42 (0.10–5.27)	4.23 (1.80–6.12)	0.01 (0.00–0.04)	<0.001
	Week 2	7.74 (5.72–9.17)	8.17 (7.36–9.35)	0.41 (0.11–2.21)	<0.001
	Week 3	7.96 (6.81–9.33)	8.10 (7.27–9.45)	3.38 (1.62–7.90)	<0.001
	Week 4	8.14 (6.98–9.41)	8.35 (7.71–9.62)	4.20 (1.93–7.94)	<0.001
	Week 5	7.70 (6.56–8.82)	8.07 (7.51–8.89)	6.41 (2.98–8.74)	0.006

## Discussion

This study was the first to clearly describe a COVID-19 outbreak event in land border area of China, which was the largest COVID-19 outbreak caused by Delta VOC in China's border areas. Because of timely control measures and proactive case detection and tracking, patients were detected and treated in a timely manner, with few cases of severe or critical illness and no deaths. The fact that nearly half of the COVID-19 patients came from Myanmar showed that this was a cross-border outbreak. Several imported outbreaks of Delta VOC have been reported in China, and most of them were related to imported cases on specific international flights, such as the outbreak in Nanjing and Guangzhou (3, 4, 11–13). The single source of infection led to the chains of transmission fewer and relatively clear in the outbreak caused by flights imported cases. The outbreak in Nanjing in July, 2021 was caused by a flight from Moscow, and the index case in China was a cabin cleaning staff member servicing the flight at Nanjing Lukou Airport (4, 11). After that, the virus spread to other cleaning staff, airport passengers, and then to local communities leading to community transmission, the transmission chain of which was relatively clear (4, 11). Similar to the outbreak in Nanjing, the outbreak in Guangzhou occurred from May 21 to Jun 18, 2021 was also due to the first confirmed local case's accidental exposure to an imported case (3). A clear seven-generation transmission chain including 157 patients was found based on epidemiological investigation (3). However, in stark contrast to those imported outbreaks related to international flights, the Ruili outbreak was due to imported Myanmar cases entering China through land border. Before the “July 4” COVID-19 outbreak, though the Ruili City's border management policy has not been relaxed, the newly reported COVID-19 cases in Myanmar increased sharply from late May, 2021, which could lead to an increase in the proportion of infected persons entering Ruili City from Myanmar and brought higher pressure of COVID-19 importations (Supplementary Figure 2). Therefore, because new sources of infection kept entering, the 16 transmission chains with Myanmar patients serving as index cases of several chains indicated the possibility of multi-source transmission of imported cases from Myanmar, which was also a good demonstration of the difficulties in COVID-19 control at land border ports. In addition, several transmission chains had only one case, there might be two reasons to explain this phenomenon. First, although the epidemiological investigation has been thorough, some potential contacts between the patients might not be found; and insidious transmission by probable cases who could not be detected by nucleic acid testing might exist (14), which could also make the initial transmission difficult to be observed. Second, it is possible that some imported cases were detected early under relatively strict outbreak control measures, which avoided further transmission.

Compared with the patients' characteristics in the Nanjing and Guangzhou's outbreak, more patients from Myanmar were found in the community transmission in Ruili City, while almost all of patients in Nanjing and Guangzhou's outbreak were Chinese (3, 11). Because many young adults were engaged in goods trading in Ruili City, the age of COVID-19 patients in Ruili's outbreak (34.01 ± 16.18 years) was much younger than that in the outbreak in Nanjing (43.25 ± 16.96 years) and Guangzhou (Median age 47.0 years) (3, 11). Similar with Nanjing and Guangzhou's outbreak, the majority of patients had mild and moderate symptoms, but the proportion of patients with severe or critical symptoms was much lower in Ruili's outbreak (4.3%) than Guangzhou's outbreak (12.5%), which might be due to the high proportion of elderly patients (33%) in Guangzhou's outbreak (3, 11). However, patients with mild symptoms may not be aware of their symptoms and may not seek medical services. The higher proportion of patients with mild symptoms in Ruili's outbreak than that in Guangzhou (45.3% vs. 25.8%) brought challenges to the effective implementation of symptom monitoring and was more likely to cause hidden spread in communities (3).

According to the comparison of breakthrough cases and those who did not vaccinated against COVID-19, one obvious difference was that no children infections were breakthrough cases. The reason was that China had not provided COVID-19 vaccination to minors prior to the current outbreak (15). Similar to previous study, the antibodies value rose sharply in the first 2 weeks on admission after onset of symptoms among both vaccinated and non-vaccinated patients (16). The more rapid response to infection of vaccinated patients' antibodies could be related to the presence of immune memory after vaccination; after infection of SARS-CoV-2, lymphocytes in the body would be activated and antibodies could be generated rapidly in a short time (17, 18).

This study had strong practical implications. First, it should be cautious in opening borders, especially for land borders which represented by the China-Myanmar border where the COVID-19 epidemic is under effective control on one side of the border, while the epidemic of the other side is out of control. Providing medical services and vaccinations to refugees around border areas is also beneficial to COVID-19 prevention and control in the importing countries, which could also provide references for some European countries where refugee crisis was worsening (19). Second, most of the patients in this outbreak were breakthrough cases, and previous report had indicated that the effectiveness of vaccines against infection was limited (20). Therefore, some necessary non-pharmacological interventions could not be abandoned, especially for personal protection measures, such as facemask wearing. However, a limitation of this study was that the follow-up period was relatively short, and the change of antibodies in a longer time after infection could not be reflected well. In further researches, it is necessary to strengthen the exploration of SARS-CoV-2 antibody response mechanism. In addition, it is essential to explore the similarities and differences of SARS-CoV-2 antibodies in determining the course of disease in vaccinated and non-vaccinated people, and to verify the feasibility of antibodies as biomarkers to determine the time of infection.

In conclusion, this study revealed the epidemiological and clinical characteristics of the largest COVID-19 outbreak caused by Delta VOC in the China-Myanmar border area. Different from the clear single transmission chain caused by international flights passengers in other cities' COVID-19 outbreaks, 16 transmission chains were found in the “July 4” outbreak. Patients vaccinated against COVID-19 before infection had relatively higher antibodies (IgM and IgG) levels and more rapid response to infection than non-vaccinated patients. This study indicated that attention should be paid to the control and research of imported COVID-19 cases in border areas, and research on the immune effect of COVID-19 vaccines and related mechanism should be strengthened.

## Data availability statement

The original contributions presented in the study are included in the article/Supplementary material, further inquiries can be directed to the corresponding authors.

## Ethics statement

The studies involving human participants were reviewed and approved by the Mang City Center for Disease Control and Prevention, Yunnan, China. All participants gave oral informed consent because of their level of education and the urgency of emerging infectious disease's control in accordance with the national legislation and the institutional requirements.

## Author contributions

TS and BZ had full access to all of the data in the study and takes responsibility for the integrity of the data and the accuracy of the data analysis. Concept and design: XY and TS. Drafting of the manuscript: XY, ZW, and BZ. Statistical analysis: XY, XW, XZ, and ZW. Obtained funding: ZJ and BZ. Administrative, technical, or material support: LH and LC. Supervision: TS, TL, and BZ. Acquisition, analysis, or interpretation of data and critical revision of the manuscript for important intellectual content: All authors. All authors read and approved the final manuscript.

## Funding

This work was supported by National Natural Science Foundation of China [Grant Numbers 72104008, 72174004, 91546203, and 91846302], National Key Research and Development Program of China [Grant Number 2021YFC0863400].

## Conflict of interest

The authors declare that the research was conducted in the absence of any commercial or financial relationships that could be construed as a potential conflict of interest.

## Publisher's note

All claims expressed in this article are solely those of the authors and do not necessarily represent those of their affiliated organizations, or those of the publisher, the editors and the reviewers. Any product that may be evaluated in this article, or claim that may be made by its manufacturer, is not guaranteed or endorsed by the publisher.
