# Supplementary material for: Epidemiological and clinical characteristics of the largest COVID-19 outbreak along the China-Myanmar border in Ruili City, Yunnan Province, China
Source: Front Public Health. 2022 Aug 23;10:962214. doi: 10.3389/fpubh.2022.962214 (PMC9446244; doi:10.3389/fpubh.2022.962214)
Supplement: Supplementary file 1 [file Data_Sheet_1.docx]

**Supplementary file**


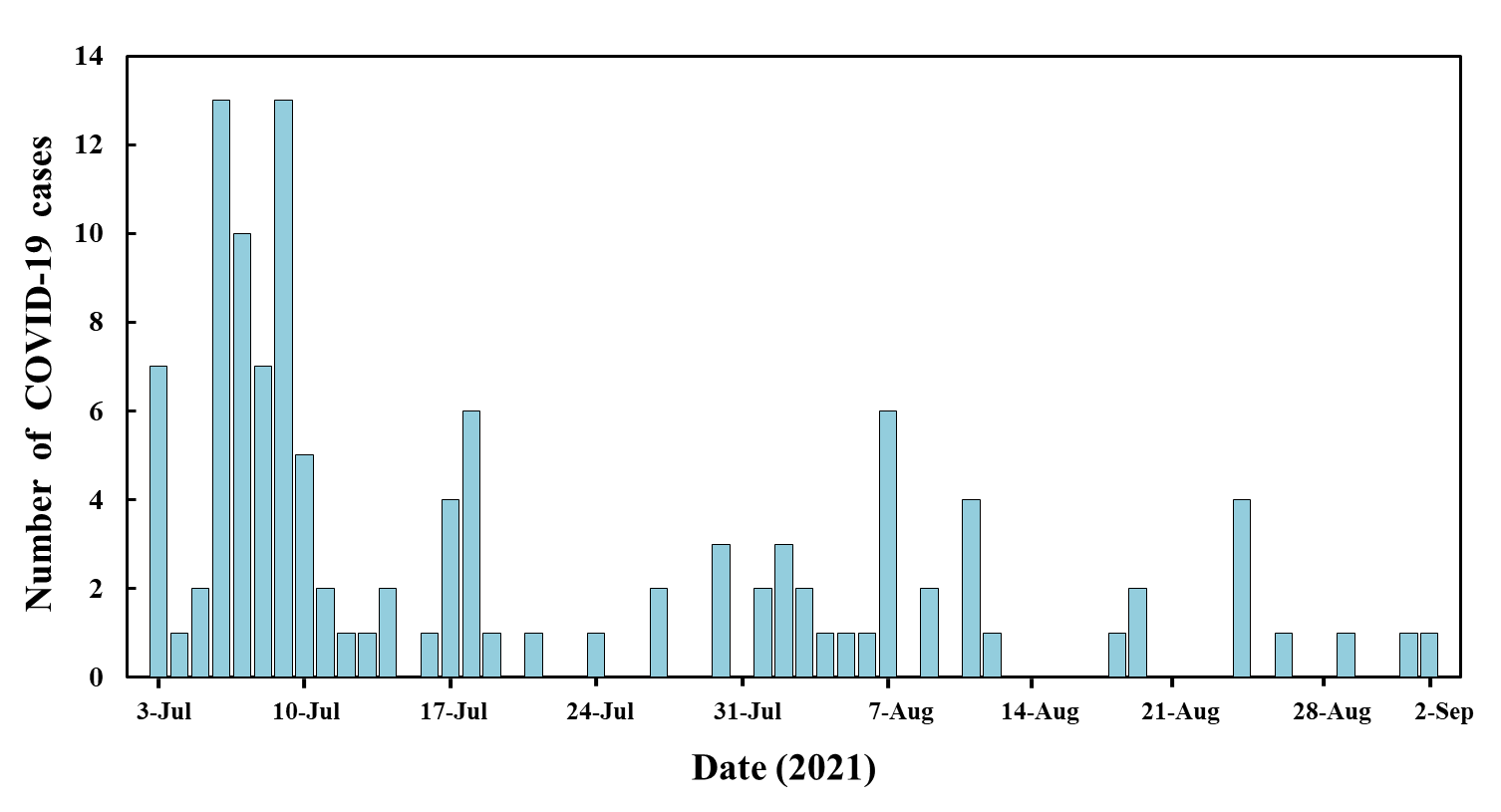


**Supplementary Figure 1. Number of patients with positive nucleic acid test result during the outbreak**

**
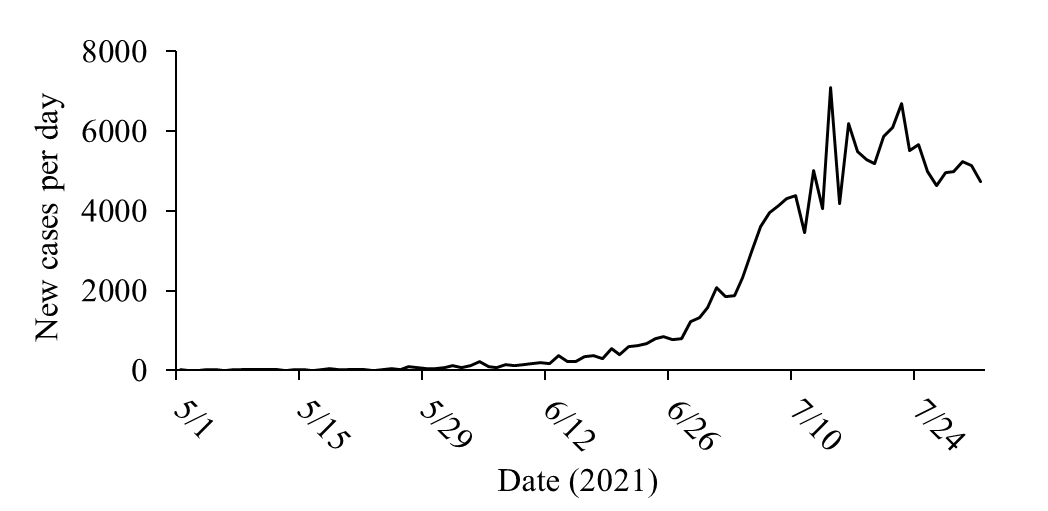
**

**Supplementary Figure 2. Number of new COVID-19 cases per day in Myanmar**

(Data source: Our World in Data—Coronavirus (COVID-19) Cases. Available at: https://ourworldindata.org/covid-cases)
